# Recovering vision in corneal epithelial stem cell deficient eyes

**DOI:** 10.1016/j.clae.2019.04.006

**Published:** 2019-08

**Authors:** Kiranjit K. Bains, Hideki Fukuoka, Greg M. Hammond, Chie Sotozono, Andrew J. Quantock

**Affiliations:** a Structural Biophysics Group, School of Optometry and Vision Sciences, Cardiff University, Maindy Road, Cardiff, Wales, United Kingdom; b Department of Ophthalmology, Kyoto Prefectural University of Medicine, 465 Kajiicho, Kamigyo-ku, Kyoto 602-8065, Japan

**Keywords:** Cornea, Corneal epithelium, Ocular surface disease, Limbal stem cell deficiency

## Abstract

A healthy corneal epithelium, which is essential for proper vision and protection from external pathogens, is continuously replenished throughout life by stem cells located at the limbus. In diseased or injured eyes, however, in which stem cells are deficient, severe ocular problems manifest themselves. These are notoriously difficult to manage and as a result the last 20 or so years has seen a number of therapeutic strategies emerge that aim to recover the ocular surface and restore vision in limbal stem cell deficient eyes. The dominant concept involves the generation of laboratory cultivated epithelial cell sheets expanded from small biopsies of the epithelial limbus (for patient or donors) or another non-corneal epithelial tissue such as the oral mucosa. Typically, cells are grown on sterilised human amniotic membrane as a substrate, which then forms part of the graft, or specially formulated plastic culture dishes from which cells sheets can be released by lowering the temperature, and thus the adherence of the plastic to the cells. Overall, clinical results are promising, as is discussed, with new cultivation methodologies and different cell lineages currently being investigated to augment the treatment options for visual disturbance caused by a corneal epithelial limbal stem cell deficiency.

## Highlights

• Corneal limbal epithelial stem cells deficiencies cause severe ocular surface instability and visual impairment.
• These conditions, caused by injury or disease, are very difficult to treat.
• Laboratory-grown epithelial cell sheets expanded from healthy limbal tissue can be used to reconstruct the ocular surface.
• Other epithelia, such as the oral mucosa, can be used to generate the therapeutic cell sheets.

## 1 Introduction

A healthy corneal epithelium is essential for vision. Patients often present with conditions that impact negatively on the integrity of the ocular surface and these can include minor abrasions, allergies, keratitis, dry eye, ocular herpes, and pterygium to name just a few. Treatment regimens for these conditions often involve a pharmacological approach. However, when the epithelium is defective owing to limbal stem cell deficiency, treatment is notoriously difficult. Corneal epithelial stem cells are widely believed to reside in the basal epithelium at the limbus [1,2]. Certainly, not all epithelial cells in the limbal basal epithelium are stem cells, and whilst there seems to be no agreed consensus as to what proportion might be stem cells in the healthy eye, it is reasonable to conclude that there must be sufficient numbers to counteract the loss of superficial central corneal epithelial cells into the tear film. As described elsewhere [3,4], the corneal epithelium is able to be continually replenished because stem cells undergo asymmetric cell division, producing two daughter cells; one remains as a stem cell whilst the other becomes a transient amplifying cell. In the corneal epithelium these transient amplifying cells migrate inwardly over the corneal surface from the limbus, becoming fully differentiated corneal epithelial cells as they do so [2]. But, in the absence of an adequate number of limbal stem cells to act as a reservoir for the corneal epithelium, the ocular surface becomes severely compromised. Some contemporary approaches to treat vision loss caused by corneal epithelial stem cell deficiency exist and will be described as follows.

## 2 Corneal epithelial limbal stem cell deficiencies

A corneal epithelial limbal stem cell deficiency can arise because of a thermal or chemical burn, extensive mechanical trauma, inflammatory disease, contact lens wear, and/or iatrogenic trauma [5,6]. Clinically, it can be manifested by the presence of corneal neovascularisation, chronic inflammation, conjunctivalization, and/or a persistent epithelial defect. Importantly, autoimmune conditions such as Stevens-Johnson syndrome and ocular cicatricial pemphigoid exhibit severe ocular surface pathology related to a limbal epithelial stem cell deficiency and have links to infectious keratitis [7]. A recent ten-year retrospective analysis of 1331 patients with limbal epithelial stem deficiencies in two major eye hospitals in India by Vazirani and associates [8] reported that most patients presented with a unilateral stem cell deficiency (791 patients versus 540 with bilateral deficiencies) and that ocular surface burns were the most common identifiable cause of these at almost 85%. Exposure to lime, used in the construction industry, caused over 60% of these injuries, but this is likely to be lower in the UK because of more stringent safety practices. The leading identifiable causes of bilateral stem cell deficiencies, on the other hand, were ocular surface burns (30%), allergic conjunctivitis (29%), Stevens-Johnson syndrome or toxic epidermal necrolysis (23%), aniridia (9%), and mucous membrane pemphigoid (4%).

**Fig. 1:**
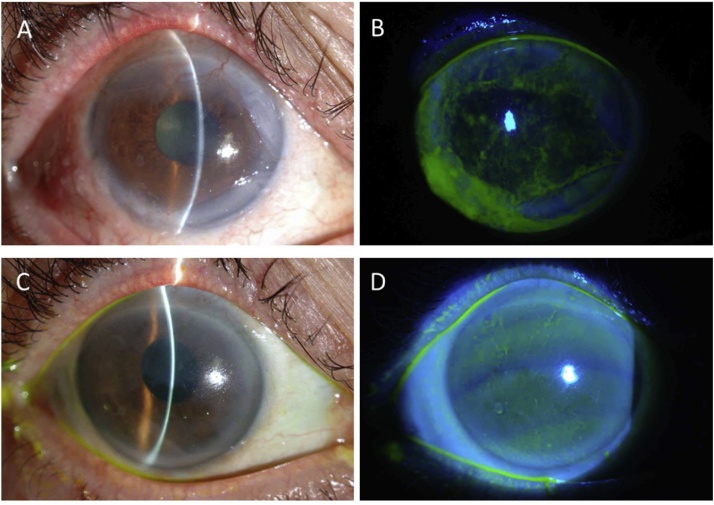
Clinical photographs of the patient's eye before (A,B) and after (C,D) allogenic cultivated limbal epithelial transplantation (CLET). Ocular surface squamous neoplasia (OSSN) recurred and covered the entire corneal surface at 6-years post tumor resection (A). Fluorescein staining of the same eye showed an irregular ocular surface (B). CLET was performed combined with tumor resection, and phacoemulsification and intraocular lens transplantation (PEA + IOL). At 1-year post CLET, the cornea was covered by healthy corneal epithelial cells derived from the transplanted cultured corneal epithelial cell sheet (C). Fluorescein staining showed a smooth and stable corneal surface (D). Best-corrected visual acuity (BCVA) improved from 0.15 to 0.7.

**Fig. 2:**
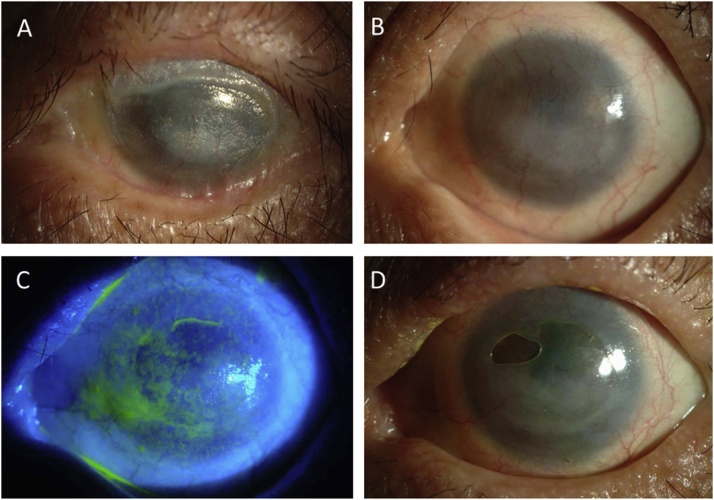
Clinical photographs of the patient's eye before (A) and after (B,C,D) autologous cultivated oral mucosal epithelial transplantation (COMET). COMET was performed combined with keratectomy, amniotic membrane transplantation, and PEA + IOL for end-stage ocular pemphigoid with keratinization and symblepharon (A). Symblepharon was successfully released, and non-keratinized epithelium derived from the cultivated oral mucosal epithelial cell sheet covered the entire corneal surface at 3-years post COMET (B), and BCVA improved from hand motion to count fingers post COMET. Fluorescein staining of the same eye post-COMET showed no epithelial damage (C). COMET enabled the use of a tear-exchangeable, limbal, rigid CL, and BCVA improved to 0.05 via the use of this CL (D).

It is important for the clinician to note that individuals with compromised limbal stem cell function often present with symptoms that include reduced vision, photophobia, and varying degrees of ocular discomfort [5,[9], [10], [11]]. Conjunctivalization of the ocular surface also occurs [12], and this can be recognised upon slit lamp examination by the appearance of varying amounts of superficial neovascularisation, corneal opacification/scarring and, on occasion, a reduced tear film break up time [5,6,11]. The conjunctival epithelium that covers the cornea is more permeable than the corneal epithelium [13], and this can lead to a characteristic “late” fluorescein staining of the cornea, when the staining is not detected initially after fluorescein instillation, but is observed 10–15 minutes later [5,9]. Whatever the underlying cause(s) of a corneal limbal epithelial stem cell deficiency treatment is extremely challenging, with early identification and referral is likely to be beneficial to access new surgical approaches that are helping to address the problem (Figs. 1and 2).

## 3 Ocular surface reconstruction in limbal stem cell deficient eyes

Over the past two decades or so since the pioneering work of Pellegrini, Tsai, Kinoshita and others, the innovative use of limbal transplantation techniques using allogeneic or autologous tissue sources -- that is, respectively, cells that are foreign to the patient (donor-derived), or cells that are secured from the patient themselves – has shown considerable promise for the cultivation of epithelial stem cells to generate cell-based constructs for the reconstruction of the ocular surface [[14], [15], [16]]. The underlying concept is that the transfer of *ex vivo* expanded epithelial multi-layers onto the eye will repair and regenerate the stem cell deficient corneal surface to facilitate renewed epithelial healing, regression of vascularization, and prevention of recurrent erosion [17,18].

Researchers have investigated several ways of generating stratified and functional corneal epithelial cell sheets. An explant culture system was among the first procedures to be used and involves the placement of a small limbal biopsy of healthy tissue onto sterilised portions of human amniotic membrane (i.e. the inner part of the human placenta), which acts as the substrate for *ex vivo* cell expansion. Corneal epithelial cells migrate from the biopsy and adhere to the amniotic membrane, which then serves as a carrier to physically support the expanded cell sheet as it is transplanted, along with the amniotic membrane substrate, onto the diseased ocular surface, once any fibrous scar tissue has been removed from the front of the diseased or injured cornea. Generally, around two-weeks cultivation in the laboratory is sufficient to obtain a stratified corneal epithelium [16,17]. Animal experiments have shown that the amniotic membrane can persist in the cornea for a significant period of time [19], but clinical experience does not tend to report a detrimental effect of the amniotic membrane remaining on the eye after ocular surface reconstructive surgery, possibly owing to the thin, almost transparent nature of the membrane itself. It is also possible that the amniotic membrane might aid the healing process via growth factors which are present within it [20]. The majority of early work expanding corneal epithelial cells involved a co-culture system, which incorporated a growth-arrested 3T3 fibroblast feeder layer underlying the amniotic membrane to help promote epithelial differentiation [21]. However, this is now seen to be sub-optimal, especially if the fibroblasts have a non-human animal origin. A modification of the explant approach is a suspension culture system, which utilizes enzymes (typically dispase and trypsin), to isolate limbal epithelial cells from the rest of the limbal biopsy to form a cell suspension [22]. These cells are then cultured, as before, until confluent cell sheets are formed after incubation for 12 or more days, which can be transplanted on to the ocular surface [23]. Based on the published literature it appears as though both the explant and cell suspension culture systems are effective tools for ocular surface reconstruction using allogenic and autologous cell/tissue sources, although the cell suspension method seems, nowadays, to be more widely employed.

Cells grown in laboratory conditions respond to the properties of the substrate upon which they are cultivated and the chemical environment provided by the culture media that sustains them. Human amniotic membrane is often used to promote the *ex vivo* expansion of corneal limbal epithelial cells and has become a stable in many studies. This is because, as with other materials such as collagen membranes, it can support epithelial growth [24], and its use is thought to help improve wound healing via a prevention of corneal scarring [16]. The amniotic membrane, itself, is basically a thin connective tissue, which is possessed of its own epithelium. Some researchers favour leaving the amniotic epithelium intact when expanding limbal epithelial cells on amniotic membrane, which is claimed to promote a corneal epithelial phenotype [25]. Other groups, in contrast, recommend its removal to likely facilitate better epithelial adherence [[26], [27], [28]], and this tends to be the common contemporary approach.

An alternative to the use of amniotic membrane as a substrate and carrier is the use of a temperature-responsive polymeric culture surface on which the corneal epithelial multi-layer can be formed. The chemistry of the polymer is such that when its temperature is reduced below a critical solution temperature, then the epithelial cell multi-layer loses adherence and is able to be readily detached and used, carrier-free, to reconstruct the exposed corneal stroma from which fibrotic tissue has been removed [29,30]. This approach has the perceived advantage of allowing direct interaction between the expanded epithelial multi-layer and the recipient’s ocular surface, without an intervening carrier construct. In addition to intact and denuded amniotic membrane and temperature-responsive polymers a number of other viable substrates for the ex vivo expansion of epithelial cell multi-layers, such as collagen- and fibrin-based carriers, have also been investigated for ocular surface reconstruction and reviews of these can be found elsewhere [31,32].

The nutrient media with which the expanded epithelial cells are nourished is also important for their growth and differentiation. Often, both explant and cell suspension cultivation protocols have used xenobiotic (i.e. cross-species) materials, such as murine-derived feeder cells and bovine serum. Indeed, over the past two decades, the use of foetal calf serum and murine-derived 3T3 fibroblasts, combined with Dulbecco’s Modified Eagle Medium, has become routine in the majority of approaches to refine techniques to expand epithelial cells into transplantable tissues. The use of non-human materials, however, has raised safety concerns regarding potential transmission of cross-species viruses and prions, and as a consequence, experiments have been conducted in an attempt to determine alternative xenogeneic-free substitutes to improve upon the established culture systems. Nakamura and associates, for example, investigated the efficacy of autologous human serum as a feasible replacement for fetal bovine serum and, encouragingly, found it to have equivalent results [33]. Kolli and co-workers also achieved successful expansion of epithelial cells with the use of a non-animal derived serum for the treatment of chemical burns to the ocular surface [34], whilst Sangwan and associates generated autologous cultivated limbal epithelial sheets using xeno-free explant culture techniques to treat unilateral total limbal stem cell deficiency and achieved an overall success rate of 71%, with a better than 60% improvement in visual acuity [35]. Most recently, Nakamura and co-workers were able to develop a novel feeder-free and serum-free technique to engineer transplantable tissue for the treatment of the dysfunctional ocular surfaces of rabbits that survived for up to two weeks [36]. Collectively, these advances represent key steps towards generating laboratory-grown cell constructs to treat ocular surface disease. But, what are the surgical techniques currently employed?

## 4 The cell source for corneal epithelial reconstruction using *ex vivo* expanded cell constructs

At the present time, the two most common forms of ocular surface reconstruction using expanded epithelial multi-layers are Cultured Limbal Epithelial Transplantation (CLET) and Cultured Oral Mucosal Epithelial Transplantation (COMET). In the case of CLET autologous and allogeneic cells are utilised, whereas in the case of COMET only autologous cells are employed. It is evident from the published literature that both CLET and COMET are associated with favourable clinical outcomes and based on current knowledge it is difficult to judge one approach to be manifestly superior to the other. Generally, COMET is performed for the end-stage severe ocular surface diseases such as Stevens Johnson syndrome, ocular cicatrical pemphegoid or a chemical/thermal burn to release symblepharon and/or replace severely damaged ocular surface cells. It is worth noting that the use of tear exchangeable rigid contact lenses was able to improve the visual outcome following COMET [37].

### 4.1 Cultured limbal epithelial transplantation (CLET)

For allogeneic CLET, limbal stem cells are obtained from tissue biopsied from either a living relative or donor eye, with the small tissue biopsy extending about 1 mm either side of the corneoscleral junction [38]. After the graft, vigilant postoperative care with long-term immuno-suppressants is required to avoid immunological rejection of transplanted epithelium [39], notwithstanding the risk of associated complications. The alternative is to use autologous limbal stem cells, which, of course, is contingent on the availability of healthy limbal tissue in an unaffected eye, and thus is only an option for the treatment of unilateral ocular surface disorders. As seen in Table 1, numerous articles have described the use of allogeneic and autologous stem cell sources to expand *ex vivo* limbal epithelial cells to repair the ocular surface [22,[40], [41], [42]]. Although post-operative complications following CLET can occur and have to be guarded against [23,27,[43], [44], [45]], it represents a viable method of treating severely damaged limbal stem cell deficient eyes.

**Table 1:** Summary of Published Studies Using Limbal Epithelial Stem Cells.

ARTICLE (ref)	NUMBER OF EYES/Px	AUTO/ALLO-GRAFT	TISSUE TYPE	FEEDER CELL LAYER	SUBSTRATE	PATHOLOGY	MEAN FOLLOW UP (MONTHS)	VA IMPROVEMENT*	SUCCESS RATE*
Tsubota et al. 1999 [70]	43/39	Allo	LESC	N	AM	SJS/OCP 25 Therm/Chem 14	28.2	60%	–
Schwab et al. 2000 [43]	14/14	Auto 10 Allo 4	LESC LRD	Y	D-AM	NS	13	–	71.4%
Tsai et al. 2000 [15]	6/6	Auto	LESC	N	AM	Chem 3 Other 3	15	83%	–
Koizumi et al. 2001 [27]	13/11	Allo	CAD	Y	D-AM	SJS 7 OCP 2 Chem 3 Other1	11.2	–	–
Grueterich et al. 2002 [71]	1/1	Auto	LESC	N	AM	Chem 1	21	–	–
Shimazaki et al. 2002 [44]	13/13	Allo	CAD 7 LRD 6	Y	AM	SJS 8 OCP 3 Chem 2	NS	–	46.2%
Sangwan et al. 2003 [72]	2/1	Auto	LESC	Y	D-AM	Chem 1	12	–	–
Nakamura et al. 2004 [73]	1/1	Auto	LESC	Y	D-AM	Chem 1	19	–	100%
Daya et al. 2005 [23]	10/10	Allo	CAD 9 LRD 1	Y	AM	SJS 3 Therm/Chem 3 Other 4	28	40%	70%
Nakamura et al. 2006 [33]	9/9	Auto 2 Allo 7	LESC CAD	Y	D-AM	SJS 2 OCP 1 Chem 1 Aniridia 1 Idiopathic 2 Other 2	14.6	100%	100%
Sangwan et al. 2006 [74]	88/86	Auto	LESC	N	D-AM	Therm/Chem 78 Other 10	18.3	–	73.1%
Ang et al. 2007 [75]	2/1	Allo	CAD	Y	D-AM	SJS 1	NS	–	–
Fatima et al. 2007 [76]	1/1	Auto	LESC	Y	AM	Chem 1	37	–	60%
Shimazaki et al. 2007 [77]	27/27	Auto 7 Allo 20	LESC CAD 12 LRD 8	N Y	D-AM AM	SJS 13 OCP 4 Therm/Chem 9 Other 1	29.2	48.1%	59.3%
Kawashima et al. 2007 [78]	6/6	Auto 2 Allo 4	LESC LRD 1 CAD 3	Y Y	D-AM	Chem 3 SJS 2 Pseudo-OCP 1	6.8	–	–
Shortt et al. 2008 [79]	10/10	Auto 3 Allo 7	LESC CAD	N	AM	Chem 4 Aniridia 3 ED 1 Reiger’s Anomaly 1 Other 1	13	–	60%
Kolli et al. 2009 [80]	8/8	Auto	LESC	Y	AM	NS	19	62.5%	100%
Meller et al. 2009 [81]	1/1	Allo	LRD	NS	AM	Other 1	31	–	–
Satake et al. 2009 [82]	1/1	Auto	LESC	Y	D-AM	Other 1	43	–	–
Baradaran-Rafii et al. 2010 [83]	8/8	Auto	LESC	N	D-AM	Therm/Chem 8	34	–	–
Thanos et al. 2010 [84]	1/1	Auto	LESC	N	AM	Other 1	28	–	–
Sangwan et al. 2011 [35]	200/200	Auto	LESC	N	D-AM	Therm/Chem 200	36	60.5%	71%
Sharma et al. 2011 [85]	50/50	Auto 34 Allo 16	LESC 34 CAD 9 LRD 7	N	D-AM	Therm/Chem 18 Other 2 NS 30	11	68%	74%
Basu et al. 2012 [86]	50/50	Auto	LESC	N	D-AM	Therm/Chem 50	27.6	76%	66%
Prabhasawat et al. 2012 [87]	19/18	Auto 12 Allo 7	LESC CAD	N	D-AM	Therm/Chem 13 SJS 1 Other 5	26.1	68.4%	73.7%
Pathak et al. 2012 [11]	9/9	Auto	LESC	N	AM	Therm/Chem 8 Other 1	11-28	–	55.6%
Sejpal et al. 2013 [88]	107/107	Auto	LESC	N	D-AM	Therm/Chem 107	41.2	54.2%	46.7%
Sharma et al. 2013 [89]	4/4	Auto	LESC	NS	D-AM	Therm/Chem 4	19.5	100%	–
Subramaniam et al. 2013 [90]	40/39	Auto	LESC	N	D-AM	Therm/Chem 36 Other 4	33	38%	–
Qi et al. 2013 [91]	42/41	Allo	CAD	Y	D-AM	Therm/Chem 41	12	–	–
Amescua et al. 2014 [45]	4/4	Auto	LESC	N	AM	Chem 2 Trauma 1 Melanoma 1	7.5	100%	100%
Qi et al. 2014 [92]	16/15	Allo	CAD	Y	D-AM	Therm/Chem15	12	–	80%
Vazirani et al. 2014 [93]	70/70	Auto	LESC	N	D-AM	Therm/Chem 64 OCP 1 Idiopathic 1 Other 4	17.5	–	–
Zakaria et al. 2014 [94]	18/12	Auto 15 Allo 3	LESC CAD 1 LRD 2	N	D-AM	Chem 7 Aniridia 2 Other 9	24	–	67%
Ramírez et al. 2015 [95]	20/19	Auto 11 Allo 9	LESC CAD	N	D-AM	Chem 7 SJS 3 Aniridia 2 Other 8	36	–	80%

AM=Human Amniotic Membrane. Allo = Allograft. Auto = Autograft. CAD = Cadaver. Chem = Chemical. D-AM = Denuded Human Amniotic Membrane. ED = Ectodermal Dysplasia. LESC = Limbal Epithelial Stem Cells. LRD = Living Relative Donor NS = Not Stipulated. OCP = ocular cicatricial pemphigoid. Px = Patient. SJS = Steven Johnson syndrome. Therm = Thermal. VA = Visual Acuity.

* Areas void of information are the result of values not explicitly being provided in the relevant journal articles.

### 4.2 Cultured oral mucosal epithelial transplantation (COMET)

To conduct cultured oral mucosal epithelial transplantation, stem cells are acquired from the interior buccal mucosal epithelium (inner cheek cells). First reported in 2004 [29,46], this approach has become something of a catalyst in the search for autologous stem cells as an alternative to limbal epithelial stem cells. This is especially important in the case of bilateral injury or disease, where no autologous limbal epithelial cells are available. To conduct the procedure, buccal mucosal epithelium is surgically extracted from the inner oral cavity and treated with dispase and trypsin to form a cell suspension. As described by Nishida and associates [29], this suspension is seeded onto a temperature-responsive cell-culture insert containing 3T3 feeder cells and incubated for two weeks to generate a cell sheet that can be released from the temperature-responsive culture surface by lowering the temperature and transplanted onto the ocular surface. As seen in Table 2, COMET has been employed by a number of groups with generally positive outcomes. A recent study by Nakamura and co-workers [47], for example, showed that oral mucosal cell sheets grown on amniotic membrane completely covered the membrane and displayed morphological features that resembled those of a normal corneal epithelium. When used surgically to treat limbal stem cell-deficient eyes, all with a pre-operative visual acuity (VA) of worse-than 20/500, the authors found a 95% improvement in VA overall. The follow-up period in this study ranged from 36–39 months, and it was the first to demonstrate the long-term effectiveness of COMET for ocular surface reconstruction. Other research, too, supports the claims of COMET as novel surgical therapy for treatment of severe ocular surface disease, especially for bilateral conditions such as ocular cicatricial pemphigoid, Steven Johnson syndrome, and chemical injury [[48], [49], [50], [51], [52]].

**Table 2:** Summary of Published Studies Using Oral Mucosal Epithelial Cells.

ARTICLE (ref)	NUMBER OF EYES/Px	AUTO/ALLO- GRAFT	TISSUE TYPE	FEEDER CELL LAYER	SUBSTRATE	PATHOLOGY	MEAN FOLLOW UP (MONTHS)	VA IMPROVEMENT*	SUCCESS RATE*
Nakamura et al. 2004 [46]	6/4	Auto	Oral	Y	D-AM	SJS 3 Chem 3	13.8	100%	–
Nishida et al. 2004 [29]	4/4	Auto	Oral	Y	TRS	SJS 1 OCP 3	14	100%	–
Ang et al. 2006 [49]	10/10	Auto	Oral	Y	D-AM	Therm/Chem 2 SJS 7 OCP 1	12.6	90%	–
Inatomi et al. 2006 [96]	2/2	Auto	Oral	Y	D-AM	Chem 1 SJS 1	22.5	–	–
Inatomi et al. 2006 [48]	15/12	Auto	Oral	Y	D-AM	Therm/Chem 6 SJS 7 Pseudo-OCP1 Other 1	20	67%	67%
Nakamura et al. 2007 [97]	6/5	Auto	Oral	NS	AM	SJS 3 Chem 3	NS	–	66.7%
Satake et al. 2008 [98]	4/4	Auto	Oral	Y	D-AM	SJS 2 Pseudo-OCP 2	6-24	–	–
Chen et al. 2009 [99]	4/4	Auto	Oral	Y	D-AM	Therm/Chem 4	22	–	–
Ma et al.2009 [100]	6/5	Auto	Oral	Y	D-AM	Therm/Chem 5	29.6	–	–
Nakamura et al. 2010 [47]	19/17	Auto	Oral	Y	D-AM	Therm/Chem 1 SJS 11 OCP 4 Other 3	55	95%	–
Priya et al. 2011 [101]	10/10	Auto	Oral	Y	D-AM	SJS 1 Chem 9	18.6	–	–
Takeda et al. 2011 [102]	3/3	Auto	Oral	Y	D-AM	Therm/Chem 3	30	–	–
Burillon et al. 2012 [103]	26/25	Auto	Oral	Y	TRS	Therm/Chem 9 Aniridia 3 Other 14	12	–	64%
Chen et al. 2012 [104]	6/6	Auto	Oral	Y	D-AM	Therm/Chem 6	36.7	–	–
Sotozono et al. 2013 [52]	46/40	Auto	Oral	Y	AM	Therm/Chem 7 SJS 21 OCP 10 Other 8	28.7	48%	–
Kolli et al. 2014 [34]	2/2	Auto	Oral	N	AM	Chem 2	24	–	–
Sotozono et al. 2014 [51]	10/9	Auto	Oral	Y/N	D-AM	Therm/Chem 5 SJS 3 OCP 2	23.3	–	–
Prabhasawat et al. 2016 [50]	20/18	Auto	Oral	N	D-AM	Chem/Therm 7 SJS 10 Others 3	31.9	70%	75%

Allo = Allograft. AM=Human Amniotic Membrane. Auto = Autograft. CAD = Cadaver. D-AM = Denuded Human Amniotic Membrane. Therm = Thermal. Chem = Chemical. LRD = Living Relative Donor. NS = Not Stipulated. OCP = ocular cicatricial pemphigoid. Px = Patient. SJS = Steven Johnson syndrome. TRS = Temperature Responsive Surface. VA = Visual Acuity.

* Areas void of information are the result of values not explicitly being provided in the relevant journal articles.

## 5 Future potential therapies and cell sources

As mentioned, research with non-corneal stem cells, such as those of the oral mucosa, has been a catalyst for new and exciting investigations into potential alternative non-ocular cell sources for the treatment of ocular surface dysfunction. Investigations of some of these proposed alternatives are described below.

### 5.1 Murine vibrissal hair follicle bulge-derived stem cells

Hair follicle bulge-derived stem cells can be isolated and cultured fairly readily [53]. Indeed, investigators have demonstrated the transdifferentiation potential of murine vibrissal hair follicle bulge-derived stem cells as a potential autologous stem cell source for ocular surface repair, based on their ability to assume corneal epithelial-like properties when exposed to a corneal limbus-specific microenvironment *in vitro* [54]. Subsequent work found that these hair follicle-derived cell constructs could be used to reconstruct damaged corneal epithelia in mice [55]. Specifically, the research found a significant reduction in corneal fluorescein staining four weeks after receiving the bulge-derived stem cell graft when compared to control mice not having received the transplant. This suggests that the bulge-derived stem cells were able to re-form tight intercellular junctions for successful corneal resurfacing. Similarly, the authors reported that eyes receiving bulge-derived stem cell transplants had close phenotypic resemblance to that of normal corneal epithelium, averaging 3–5 cellular layers of differentiated cells. They established an 80% success rate in graft transplantation. Twenty percent of eyes developed a minor degree of conjunctivalisation near the peripheral aspects of the cornea, however, with a 5-week follow-up period, no graft rejection was observed. These results suggest that hair follicle bulge-derived stem cells have the potential to reconstruct the corneal surface in a limbal stem cell deficiency murine model, but further investigation is required to refine and improve the overall efficacy of the cell source for clinical application.

### 5.2 Human immature dental pulp stem cells

Human deciduous teeth express markers for mesenchymal stem cells, embryonic stem cells, and limbal stem cells. Monteiro and co-workers cultured human immature dental pulp stem cells to produce a well-formed corneal epithelium, which was capable of restoring corneal clarity and smoothness in a rabbit model of corneal epithelial dysfunction and forming an epithelium that exhibited a comparable morphology to that of the normal corneal epithelium [56,57].

### 5.3 Umbilical cord stem cells

Umbilical cord stem cells have gained attention in the field of regenerative medicine because, as well as being multipotent, they are immunologically naïve, thus reducing the risk of possible infection and immunological rejection. Studies by Reza and associates used umbilical cords from healthy women undergoing delivery to cultivate umbilical cord stem cell-derived transplantable sheets for grafting onto the damaged eyes of rabbits. Eyes transplanted with umbilical cord stem cells cultivated on human amniotic membrane, compared to denuded human amniotic membrane, recovered a smooth and clear corneal surface with a multi-layered epithelium and minimal opacification and neovascularisation [58,59].

### 5.4 Embryonic stem cells

Embryonic stem cells are pluripotent cells with the ability to develop into more than 200 different cell types that make up the adult human body. They derive from epiblast tissue of the inner cell mass of a blastocyst and when given the necessary stimulation, specific cell-type differentiation is initiated. Initial studies applying embryonic stem cells to ocular surface reconstruction by Homma and associates reported grafts of expanded embryonic stem cells onto the damaged corneas of six mice [60]. Comparing the transplant-recipient group to the control group found expression of cytokeratin 12, a specific marker for corneal epithelial cells, and Pax-6, a transcriptional factor which is necessary for development of the eye. Eyes chemically damaged with n-heptanol showed complete epithelial loss, but within 24 h of transplantation, complex layering consisting of all epithelial cell types was observed across the entirety of the damaged area. These findings acted as a foundation for investigations of embryonic stem cell-derived sheets for ocular surface reconstruction. Subsequent work by Suzuki and colleagues in animal models has shown how corneal epithelial cells can be induced from embryonic stem cells, either by culturing them on type IV collagen or by introduction of the Pax6 gene into the embryonic stem cells, and demonstrated that cells survive when transplanted onto the cornea [61,62]. One of the characteristics of embryonic stem cells is that they continue to divide *in vitro*, with each daughter cell remaining pluripotent if there are no stimuli for differentiation. The regenerative potential is thus clear, however, a major hurdle to the use of embryonic stem cells in regenerative medicine is the valid ethical controversy involved with obtaining cells from extra-fertilised ova used in *in vitro* fertilisation (IVF) therapy. Many nations have a moratorium on either the production of embryonic stem cell lines or embryonic stem cell research in general, restricting work in this area [63].

### 5.5 Induced pluripotent stem (iPS) cells

Owing to the ethical concerns surrounding the use of embryonic stem cells, interest has turned to the possible use of iPS cells in regenerative medicine, and the eye is no exception. Indeed, the world’s first transplantation of iPS cells into a human was via the implantation of an autologous iPS cell-derived retinal pigment epithelium into the eye of a patient with advanced neovascular age-related macular degeneration [64]. iPS cells are mature cells that have been reprogrammed from their differentiated state to an embryonic-like state by transfer of nuclear contents [65]. Four transcription factors (known as Yamanaka factors) are capable of generating iPS cells, which exhibit the morphology and growth properties of embryonic stem cells and express embryonic stem cell marker genes. It was a pivotal discovery that led to the award of the 2012 Nobel Prize in Physiology or Medicine to Professors Shinya Yamanaka (Kyoto) and John Gurdon (Cambridge). Contemporary research with human iPS cells has provided encouragement for their potential use in the treatment of eye disease. As well as the aforementioned studies on retinal pathology [64], the discovery that human iPS cells could spontaneously form concentric colonies of cells with ocular characteristics has opened the door to future advances in the treatment of other parts of the eye, especially the corneal epithelium, with animal models already showing how a functional human iPS cell-derived epithelial cell sheet can repair severely damaged limbal stem cell-deficient eyes [66,67].

### 5.6 Mesenchymal stem cells

Mesenchymal stem cells are non-hematopoietic multipotent cells derived from bone marrow. They have been used in studies such as those by Ma and colleagues to examine whether or not human mesenchymal stem cells could successfully reconstruct the damaged ocular surface and if grafted mesenchymal stem cells could differentiate into corneal epithelial cells [68]. After isolating and expanding mesenchymal stem cells on amniotic membrane, grafts were transplanted onto chemically damaged corneas of rats and visual function measured using an optokinetic head-tracking instrument along with a slit-lamp assessment to determine the corneal status. This research found that of 51 eyes undergoing various treatments (i.e. with mesenchymal stem cells, limbal stem cells, fibroblasts, amniotic membrane alone, dexamathazone, and gentamicin), 16 eyes transplanted with mesenchymal stem cells on amniotic membrane significantly improved the damaged corneal surface and resulted in improved vision compared to the other treatments. An immunofluorescence analysis, however, failed to detect the expression of corneal epithelial specific cytokeratin K3 in the epithelia of eyes transplanted with human mesenchymal stem cells on amniotic membrane, suggesting that the efficacy of this treatment was not dependent on the differentiation of mesenchymal stem cells into epithelial cells, but was due, in part, to the expression of CD45 and interleukin 2 (glycoproteins expressed on a majority of bone-marrow derived cells), which contributed to the inhibition of inflammation and inflammation-related angiogenesis. More recently, it has been shown that sufficient stratification and expression of cytokeratins, growth factors, and tight junction proteins could be achieved in cultivated sheets obtained from limbal epithelial cells if mesenchymal cells were used as feeder cells [69], highlighting the versatility of the various cell-based therapeutic concepts and the need for a carefully thought-out strategy to achieve optimal ocular surface repair, possibly using different cells, media and substrata in combination.

## Disclosure

The authors report no conflicts of interest and have no proprietary interest in any of the materials mentioned in this article.
